# Necrology

**Published:** 1902-08

**Authors:** 


### NECROLOGY.

DR. W. T. MILLER, of Apollo, Pa., died June 18, 1902. He was a graduate of the American Veterinary College, class of 1887, a member of the American Veterinary Medical Association and the Pennsylvania State Veterinary Medical Association.

